# Remuneration for the Loss of Animals Slaughtered on Account of Contagious Diseases

**Published:** 1885-01

**Authors:** 


### REMUNERATION FOR THE LOSS OF ANIMALS SLAUGHTERED ON ACCOUNT OF CONTAGIOUS DISEASES.

This is one of the most difficult problems to solve of the many which come up in connection with the stamping out of diseases of the above nature.

Beginning with existing conditions, such diseases may be logically divided into two great classes:

1st.—Domesticated diseases, or such as have prevailed among our animals to a variable extent for an indefinite period, no one being responsible for their introduction. Texas Fever and Glanders illustrate this class.

2.—Imported diseases which find or have found their admittance into and extension over the country through the ignorance of our legislative authorities, or through the carelessness or unfitness of veterinary or other officials.

Of this class are pleuro-pneumonia, foot and mouth disease, the genuine—not the Kansas—rinderpest, or any other diseases which prevail in other countries, but are not at present, or have only recently become, domesticated in the United States.

Let us take the contagious lung plague and the foot and mouth disease for especial consideration, as they have both attracted much public interest during the past year.

They have both gained access to our shores by the carelessness or incompetency of the Washington Government through its representative officials.

With reference to the eruption of the foot and mouth disease in Maine during the spring and winter of 1884, which was so ably stamped out by the State authorities, under the guidance of our colleague, Dr. Bailey, there is not a particle of doubt that it was due to the carelessness and incompetency of the empirical Government Inspector. We would impress it on the mind of the editor of the *Drover's Journal* that this person was not, and is not, what the educated world recognizes as a veterinarian. The question then arises, who should pay the losses caused to the owners of domestic cattle from a disease thus acquired from imported cattle, which, contrary to the very rules of the government, were allowed to be driven over the public ways by the officials of the government—its veterinary inspector and Collector of Customs. Unquestionably, we say, the United States Government should be held responsible for such damages.

In the previously mentioned disease the question of damage is largely one of loss of use, but in other diseases we have to do with the question of absolute slaughter.

We will now turn our attention to the lung plague. Here we have to do with absolute slaughter. Here, also, we come face to face with another serious complication. Admitting that its first introduction is due to negligence on the part of the Washington Government in the case in point, how did the disease get across the Alleghanies into Ohio, and who is responsible for its non-recognition in Ohio, and hence its extension to Illinois, and from thence to Kentucky? We are simply suggesting questions which must surely arise when the question of settling the damage comes up for final consideration.

To our mind the Washington Government is only responsible for damage caused by imported cattle from the time of landing in the country to the time of their being delivered at the point of final destination. This includes the quarantine. From this time they become the property of State individuals and the State authorities are responsible for their sanitary condition.

In the case of the recent outbreak in the West the question of the manner of the extension of the disease becomes a most serious and complicated problem for the various authorities to solve. As the animals are said to be more or less Jerseys, the Government is relieved of much responsibility from the fact that the lung plague is unknown among the cattle on the channel islands. Hence they must have acquired it in this country.

The next question is, Where?

The question of the individual responsibility of owners, dealers, carriers, etc., is one that will also have to be settled in a uniform manner for the whole country, or the most unpleasant complications will finally arise. Diseased cattle will be smuggled and transported into States where the laws are less vigorously executed from those where the authorities are more wide awake. Another point of immense importance is, what is value of the condemned animal?

Where the owner is blameless and the authorities the responsible party, the full value of the animal slaughtered on account of contagious disease, should be remunerated to the owner.

In this regard I find myself in opposition to ruling opinions on the Continent; nevertheless, I believe right and justice are on my side.

The question is, what is the full value? In Europe, they say, the average market price, according to the nature, whether as a draft or flesh-producing animal. What are known as fancy animals have no extra value.

We do not agree with this opinion. Fancy cattle, or animals, are imported at a risk, held at a risk, cannot be insured as such, yet are of immense benefit to the country.

We would not be controlled by the speculative fevers which frequently occur with reference to such animals. The question is settled very easily as follows:

If a Bull, what are his services as such worth per year? that is, what did he net the last year or six months in this regard? If a fancy cow, say a Jersey, what was the value of her butter or milk during the last year; and what, if pregnant, would her calf be worth when weaned?

Again comes in the question of age, and the probable life of the animal, with the value of its annual earnings?

The annual earnings should be looked upon as the interest of a certain amount of capital which would give the approximate value at the time of slaughter, with the exception of the approximate earnings, for an average duration of life.

Ordinary cattle and horses, or other animals, have their well-known market value for every day in the week. We have simply made these suggestions in order to call the attention of those interested to the very complicated nature of all these questions. To treat the subject fully would require a text-book and months of study. B.

